# Estrogen withdrawal alters cytoskeletal and primary ciliary dynamics resulting in increased Hedgehog and osteoclastogenic paracrine signalling in osteocytes

**DOI:** 10.1038/s41598-021-88633-6

**Published:** 2021-04-29

**Authors:** Ivor P. Geoghegan, Laoise M. McNamara, David A. Hoey

**Affiliations:** 1 Mechanobiology and Medical Devices Research Group, Biomedical Engineering, College of Science and Engineering, National University of Ireland, Galway, Ireland; 2 Centre for Research in Medical Devices (CÚRAM), National University of Ireland, Galway, Ireland; 3 Trinity Centre for Biomedical Engineering, Trinity Biomedical Sciences Institute, Trinity College Dublin, Dublin, D02 R590 Ireland; 4 Department of Mechanical, Manufacturing, and Biomedical Engineering, School of Engineering, Trinity College Dublin, Dublin, Ireland; 5 Advanced Materials and Bioengineering Research Centre, Trinity College Dublin & RCSI, Dublin 2, Ireland

**Keywords:** Biomedical engineering, Bone

## Abstract

Estrogen deficiency during post-menopausal osteoporosis leads to osteoclastogenesis and bone loss. Increased pro-osteoclastogenic signalling (RANKL/OPG) by osteocytes occurs following estrogen withdrawal (EW) and is associated with impaired focal adhesions (FAs) and a disrupted actin cytoskeleton. RANKL production is mediated by Hedgehog signalling in osteocytes, a signalling pathway associated with the primary cilium, and the ciliary structure is tightly coupled to the cytoskeleton. Therefore, the objective of this study was to investigate the role of the cilium and associated signalling in EW-mediated osteoclastogenic signalling in osteocytes. We report that EW leads to an elongation of the cilium and increase in Hedgehog and osteoclastogenic signalling. Significant trends were identified linking cilia elongation with reductions in cell area and % FA area/cell area, indicating that cilia elongation is associated with disruption of FAs and actin contractility. To verify this, we inhibited FA assembly via α_v_β_3_ antagonism and inhibited actin contractility and demonstrated an elongated cilia and increased expression of Hh markers and *Rankl* expression. Therefore, our results suggest that the EW conditions associated with osteoporosis lead to a disorganisation of α_v_β_3_ integrins and reduced actin contractility, which were associated with an elongation of the cilium, activation of the Hh pathway and osteoclastogenic paracrine signalling.

## Introduction

Bone undergoes a continuous cycle of formation and resorption that is mediated by osteocytes, the most abundant cell type in bone^1^. Osteocytes are complex cells with chemosensory^2–4^ and endocrine functions ^5–7^, and are regarded to be the master coordinator of loading-induced bone formation ^2,4,6,8–10^. Osteocytes mediate osteoblast and osteoclast activation through numerous secreted factors including Receptor activator of nuclear factor κB ligand (RANKL) and sclerostin, which promote osteoclast formation and inhibit osteoblastogenesis respectively, in addition to Osteoprotegerin (OPG), a decoy receptor for RANK^4,6,11,12^.

Post-menopausal osteoporosis is a disease characterised by a decrease in circulating estrogen levels and an imbalance in bone cell remodelling, which causes bone loss and an increased susceptibility to fracture^13^. Osteoblastic bone cells from osteoporotic patients exhibited an impaired osteogenic response to mechanical stress, compared to cells from healthy patients^14^. Osteocytes exposed to an in vitro cell culture regime of estrogen withdrawal, mimicking that of post-menopausal osteoporosis, displayed a greater degree of apoptosis^15^ and an attenuated Ca^2+^ response to fluid flow^16^. Estrogen deficiency induced by ovariectomy (OVX) led to increased RANKL production by osteocytes^17^ and alterations in bone volume and trabecular thickness and organisation^18,19^. Most recently, osteocytes subjected to in vitro estrogen withdrawal conditions were shown to exhibit increased *Rankl* expression^20^ and increased osteoclastogenesis^21,22^. Interestingly, it has been established that RANKL production is mediated by Hedgehog (Hh) signalling in osteoblasts and osteocytes^23,24^, a key signalling pathway associated with the primary cilium^25^. However, whether changes in RANKL expression during estrogen deficiency are associated with changes in primary cilia mediated Hh signalling is unknown.

The primary cilium is a singular non-motile microtubule-based appendage found on the surface of most mammalian cells^26–28^. It consists of nine circumferentially arranged microtubule doublets that extend from the centrosome/basal body, which in turn is connected to the cytoskeleton^29^. The cilium represents a distinct cellular microdomain with tightly controlled transport of cargo between the cytoplasm and the organelle. Due to the specific localisation of ion channels and receptors within this domain, the cilium is known to possess both mechanosensory and chemosensory functions in many cells types including osteocytes^26,30–37^. Binding of Hh ligand to the Ptch receptor initiates the translocation of Ptch from the ciliary domain and subsequent entry of the Smo protein, facilitating the activation of Gli transcription factors at the ciliary tip^38–40^. While the function of Hh signalling has not been extensively investigated in osteocytes^24^, it has been well studied in osteoblasts and other cell types, where activation of Hh signalling indirectly induces osteoclast differentiation by upregulating RANKL expression, leading to a severe osteopenic phenotype^23^. Interestingly, Hh signalling can be regulated via modulation of cilia length^41,42^. Therefore, the osteocyte primary cilium via Hh signalling may play an important role in osteoclastogenesis and bone remodelling. Therefore, the focus of this research was to understand the role of Hh signalling and the primary cilium in pro-osteoclastogenic signalling by osteocytes under estrogen deprived conditions representative of post-menopausal osteoporosis.

The primary cilium interacts with many cellular components including integrin-based adhesions^43^ and the cytoskeleton^44^. Integrins are heterodimeric transmembrane proteins, comprised of α and β subunits, which connect the extracellular matrix to the intracellular cytoskeleton at distinct focal adhesion (FA) sites^45^. The integrin α_v_β_3_ has been shown to be important in osteocytes, with β_3_ integrin sites along osteocyte cell processes were found to be key sites for osteocyte strain amplification^46–49^. Further to this, α_v_β_3_ antagonism was shown to effect osteocyte morphology and responses to mechanical stimulation^20,50,51^. The α_8_ integrin subunit has even been shown to regulate ciliogenesis in neurosensory cells^52^. Integrins are known to connect to the primary cilium via the actin cytoskeleton, but have also been shown to directly connect the basal body of the primary cilium to the actin cytoskeleton in specialised structures known as ciliary adhesions^43^. Given the fact that the primary cilium is a microtubule based appendage, it is unsurprising that the cilium and the microtubule cytoskeletal network are functionally linked^29,44^. However, other cytoskeletal proteins such as actin and septin have also been shown to play a role in primary cilium dynamics^44,53^. This was most clearly demonstrated by Pitaval et al*.*, where a spread cell shape or high substrate stiffness, both of which promoted increased intracellular tension, corresponded to a reduced cilia length, a finding that was reversed by inhibiting actin contractility using blebbistatin or Y27632^53^. Y27632 acts on the actin cytoskeleton by inhibiting ROCK-I, ROCK-II, and PRK-2 ^54^. The Rho kinases are responsible for regulating actin contractility via MLK phosphorylation with actin fibres stabilised by LIMK activation and subsequent cofilin phosphorylation ^55^, with this Rho kinase pathway sensitive to Rho-ROCK inhibitors such as Y27632 ^56^. While these studies indicate that integrins and actin contractility have the ability to regulate cilium dynamics, this relationship and potential significance has yet to be shown in osteocytes.

While the effect of OVX induced estrogen deficiency on primary cilia dynamics and associated signalling in bone cells has not been investigated, it has been shown that the deletion of ERα in mouse oviduct cells led to an increased cilia length and altered cilia-related signalling^57^. Moreover, we have shown that estrogen withdrawal in MLO-Y4 osteocytes led to a disrupted FA assembly and increased *Rankl*/*Opg* ratio^20^. Therefore, we hypothesised that estrogen withdrawal disrupts FA assembly and actin contractility, which in turn mediates a lengthening of the primary cilium. This lengthening of the cilium would result in an increase in Hh signalling and a subsequent shift to pro-osteoclastogenic paracrine signalling via enhanced RANKL expression. Specifically, we investigated the effect of estrogen withdrawal on (1) primary cilia incidence, structure, and associated Hh signalling, and (2) osteoclastogenic signalling. We also investigated the association between α_v_β_3_-containing focal adhesion assembly and the actin cytoskeleton with primary cilia lengthening following estrogen withdrawal and the effect of α_v_β_3_ antagonism and actin contractility inhibition and on primary cilia incidence, structure and associated Hh signalling, and osteoclastogenic signalling.

## Materials and methods

### Cell culture and estrogen treatment regimes

MLO-Y4 mouse osteocyte-like cells (Kerafast) were cultured on type I collagen (0.15 mg/ml in 0.02 M acetic acid and phosphate buffered saline (PBS)) coated T-75 flasks in α-minimum essential media (α-MEM) supplemented with 5% fetal calf serum (FCS: Biosera), 5% fetal bovine serum (FBS: Biosera), 2 mM l-glutamine, 100 U/mL penicillin, and 100 µg/mL streptomycin at 37 °C in a humidified environment at 5% CO_2_. The effect of estrogen treatment and estrogen withdrawal on MLO-Y4 cells was studied using the following groups: (1) continuous treatment with 10 nM 17β-estradiol for 5 days (E), and (2) pre-treatment with 10 nM 17β-estradiol for 3 days and withdrawal for 2 further days (EW), following previous approaches developed in our laboratory^16,20^. The EW group is a model of reduced estrogen levels as found in post-menopausal osteoporosis, rather than a complete removal of estrogen due to the presence of estrogen in FBS. On day 3, cells were seeded onto collagen coated substrates, and cultured for two days in accordance with their treatment groups. For immunocytochemistry experiments, on day 3 cells were detached and seeded at a density of 10,000 cells per coverslip (ø 12 mm). For PCR experiments, cells were seeded at a density of 200,000 cells per slide.

### Actin contractility inhibition

Contractility of the actin cytoskeleton was inhibited using Y27632 (Sigma)^53^. A pilot study was conducted to determine the optimal dose of Y27632 for use on MLO-Y4 osteocytes (Supplementary Fig. 2). After removal of culture medium, 1 mL of media containing 100 µM Y27632 was added to each slide for 1 h. Following this, slides were washed twice with PBS.

### Integrin α_***v***_***β***_***3***_ antagonism

The integrin α_v_β_3_ was blocked using a small molecule inhibitor for α_v_β_3_ integrins, IntegriSense 750 (PerkinElmer)^20,50,51^. After removal of culture medium, 1 mL of media containing 0.5 µM IntegriSense 750 was added to each slide for 30 min. Following this, slides were washed twice with PBS.

### Immunofluorescence

Immunofluorescence was used to study the effect of each group on primary cilium prevalence and length. Cells were also stained for actin and vinculin to understand if they played any role in the changes seen in primary cilia length. Briefly, the cells were washed in PBS and fixed in 3.8% formaldehyde solution, and then permeabilised in 0.1% Triton-X. The cells were incubated in 1% BSA to prevent non-specific binding occurring during the staining process.

Primary cilium staining was performed by staining for antibodies against acetylated α-tubulin and pericentrin to stain the ciliary body and centrioles respectively. Focal adhesions were stained using an antibody against vinculin. Secondary antibodies were used to label the proteins of interest. (See Supplementary Table 1 for detailed information on antibodies used). The cells were also stained with TRITC (Tetramethyl Rhodamine Iso-Thiocyanate) Phalloidin or FITC Phalloidin, as required, and DAPI (4′,6-diamidino-2-phenylindole) to facilitate imaging of the actin cytoskeleton and nucleus respectively. Z-stack imaging was done using an Olympus IX83 epifluorescent microscope with a 100 W halogen lamp at a magnification of 100x (N.A. 1.4, oil immersion) with a step size of 0.25 µm.

Best microscopy practices were implemented with the time between staining and imaging kept consistent for all samples. All image analysis was completed using ImageJ software^58^. The z-stacks of the images taken were combined as maximum intensity projections and these combined images were used for all image analysis. Primary cilium length was measured using a previously described method, whereby the length of the cilium was calculated by taking the projection of the cilium in the z-direction and forming a right angle triangle to capture the true length of the cilium^59^. Cell area and overall actin fluorescence intensity were measured using the actin stained images. Cell area was measured by thresholding the images to remove background fluorescence and then using the “wand tool” to select the region of interest around each cell. Where two cells were touching, the region of interest was drawn manually with the “freehand tool”. The actin fluorescent intensity was measured using the integrated density of each cell with their corresponding background integrated density subtracted, with the results presented as arbitrary units. Anisotropy of the actin fibrils was determined using a ImageJ plugin, known as FibrilTool^60^. Anisotropy results are presented as a figure between 0 and 1, whereby 0 means isotropy (actin fibres not aligned) and 1 means anisotropy (actin fibres completely aligned). The vinculin stained images were used to identify distinct focal adhesion sites across the entire cell. Identification of distinct focal adhesion sites was enabled by means of a previously published semi-automatic protocol^61^. Focal adhesion area per cell was normalised to cell area.

### Real time PCR

Relative gene expression was studied by quantitative Real Time Polymerase Chain Reaction (qRT-PCR). The genes of interest included *Gli1*, *Ptch1*, *Rankl*, and *Opg*, with *Rpl13A* used as a reference gene (Supplementary Table 2). RNA was isolated using Qiagen RNeasy kits as per manufacturer’s instructions. RNA purity and yield were assessed using a spectrophotometer (DS-11 FX, DeNovix), with 260/280 ratios of > 1.85 for all samples. 250–500 ng of RNA was then transcribed into cDNA using Qiagen Quantinova reverse transcription kits and thermal cycler (5PRIMEG/O2, Prime). qRT-PCR was carried out with a Qiagen Quantinova SYBR Green PCR kit and a StepOne Plus PCR machine (Applied Biosciences). Analysis of the results was done using the Pfaffl method^62^.

### Statistical analysis

Data is presented as mean ± standard deviation. Statistical significance was determined by means of unpaired two-tailed Student’s t-tests. Linear trends between multiple groups were determined by a post-test following a One-Way ANOVA. All statistical analyses were performed using GraphPad Prism version 6 (Windows, GraphPad Software, La Jolla California USA, www.graphpad.com) and p-value of 0.05.

## Results

### Estrogen withdrawal resulted in an elongation of the primary cilium

**Figure 1:**
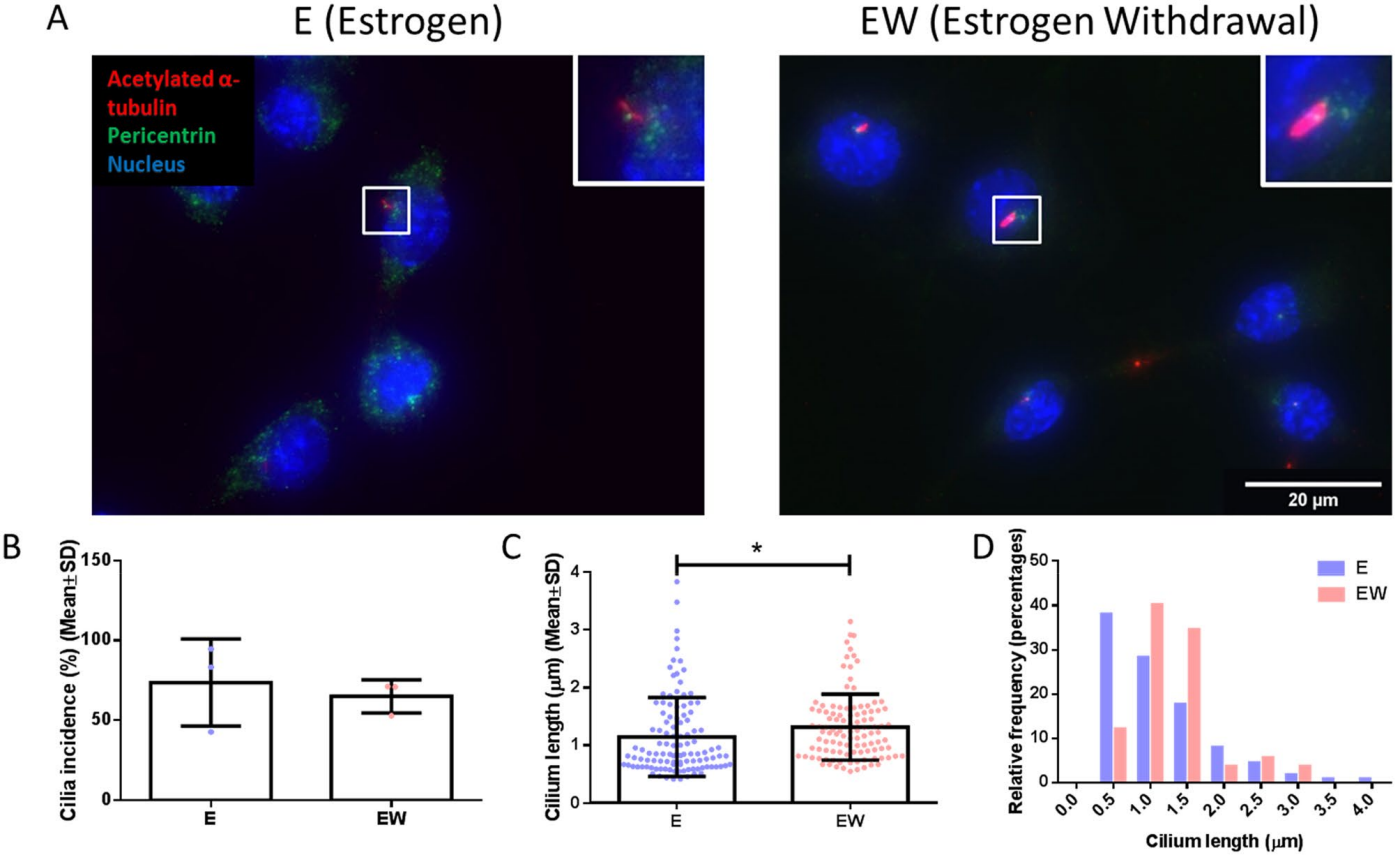
The effect of estrogen withdrawal on MLO-Y4 primary cilium incidence and length. (A) Immunocytochemistry images showing acetylated α-tubulin, pericentrin, and nuclei staining (N = 3, n ≥ 107 cells per group). Quantification of the images showing (B) primary cilium incidence, (C) primary cilium length, and (D) distribution of primary cilia lengths. (Student’s t-test, *p < 0.05).

Cells cultured under both estrogen and estrogen withdrawal conditions possessed primary cilia, as confirmed by positive staining for acetylated alpha-tubulin characteristic of a cilia-like morphology extending a length from a pericentrin-positive basal body (Fig. 1A). Quantification of the images revealed that there was no statistical difference in primary cilia prevalence between both conditions (Fig. 1B). However, a significant 17.1% increase in the primary cilium length was identified following estrogen withdrawal (1.341 ± 0.625 µm), when compared to estrogen controls (1.145 ± 0.682 µm) (p < 0.05, N = 3, n ≥ 107 cells per group) (Fig. 1C). When the distribution of cilium lengths was displayed on a frequency histogram, a clear shift to increased cilium length could be seen to occur in the estrogen withdrawal cells, compared to the estrogen cells (Fig. 1D). To determine whether this change in cilia structure could be attributed to proliferation, we quantified cell number and found no differences between E and EW groups (Supplementary Fig. 1).

### Estrogen withdrawal influences cilia-associated and osteoclastogenic signalling

Given the lengthening of the primary cilium following estrogen withdrawal, we next wished to understand the effect of estrogen withdrawal on primary cilium-associated signalling and how this may influence downstream osteoclastogenic paracrine signalling.

**Figure 2:**
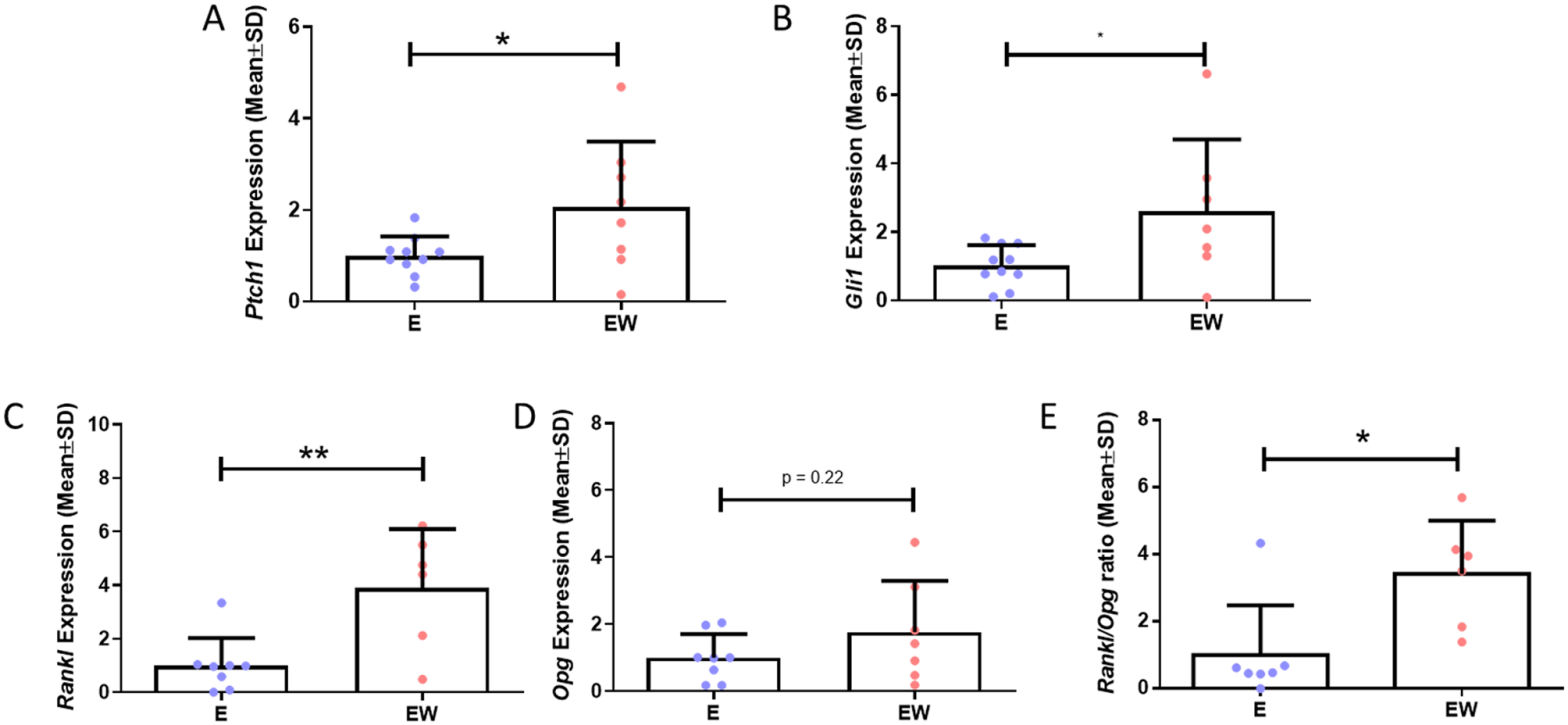
The effect of estrogen withdrawal on Hh and osteoclastogenic signalling. qRT-PCR results of (A) *Ptch1* expression (N = 8–10), (B) *Gli1* expression (N = 9–10), (C) *Rankl* expression (N = 6–8), (D) *Opg* expression (N = 7–8), and (E) *Rankl*/*Opg* ratio (N = 6–7). (Student’s t-test, *p < 0.05, **p < 0.01).

First, we investigated the effect of EW on Hh signalling markers, *Ptch1* and *Gli1*. A significant twofold increase in *Ptch1* (p < 0.05, N = 8–10) and 2.6-fold increase in *Gli1* (p < 0.05, N = 7–10) gene expression was reported following estrogen withdrawal, when compared to cells continually treated with estrogen (Fig. 2A,B).

Next, we measured markers of osteoclastogenic paracrine signalling, *Rankl* and *Opg*. We saw that *Rankl* expression was significantly higher following estrogen withdrawal (3.9-fold, p < 0.01, N = 6–8), which is consistent with previous findings^20^. However, there was no significant change in *Opg* expression (N = 7–8) (Fig. 2C,D). As such, there was an increase in the *Rankl*/*Opg* ratio following estrogen withdrawal (p < 0.05, N = 6–7) (Fig. 2E). Taken together, this data demonstrates that estrogen withdrawal results in the activation of Hh signalling and a shift towards pro-osteoclastogenic paracrine signalling by MLY-O4 osteocytes.

### Estrogen withdrawal disrupts focal adhesion assembly and intracellular actin contractility which is associated with an elongation of the primary cilium

**Figure 3:**
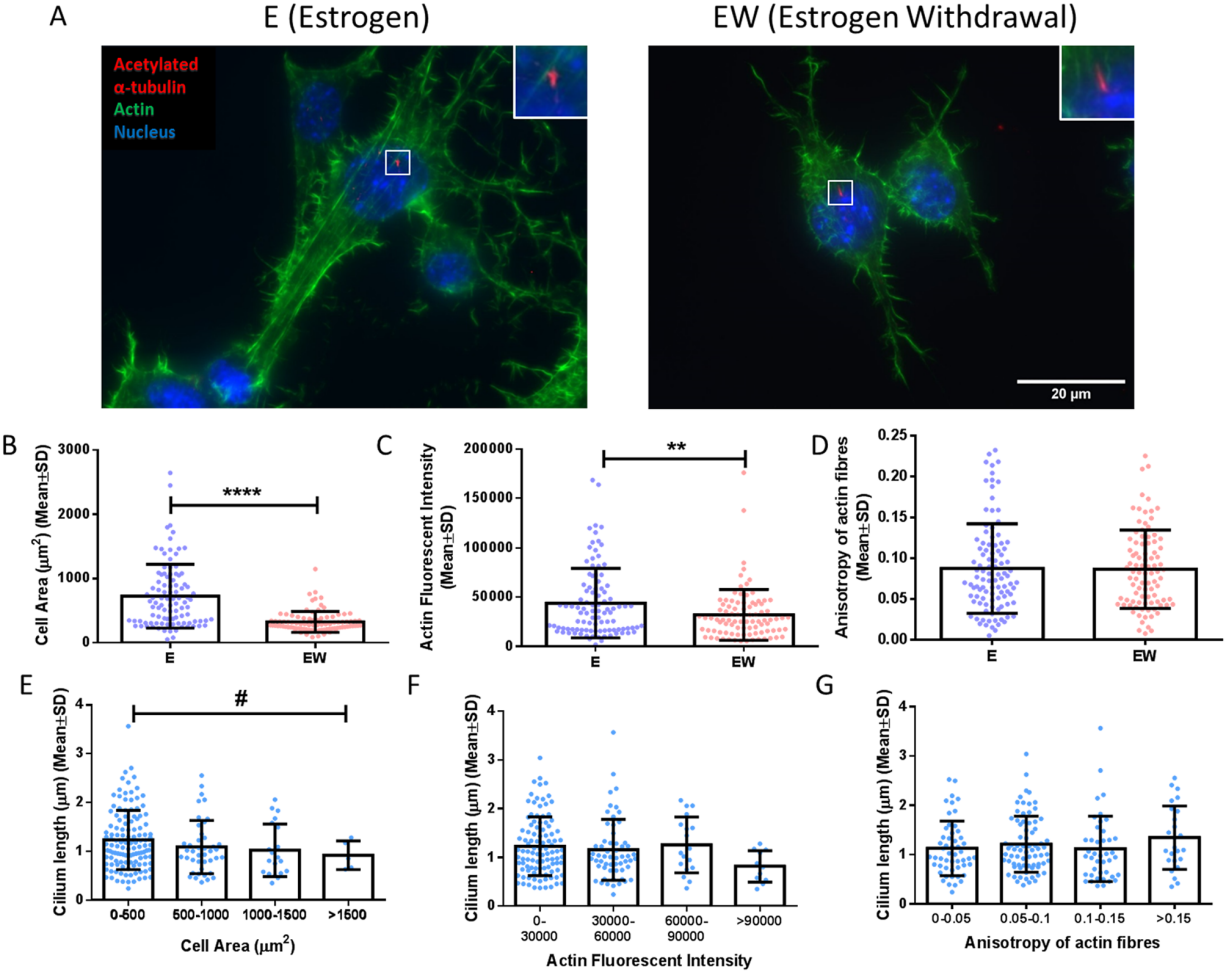
The effect of estrogen withdrawal on the role of the actin cytoskeleton in primary cilium dynamics. (A) Immunocytochemistry images showing acetylated α-tubulin, actin cytoskeleton, and nuclei staining (N = 3, n ≥ 92 cells per group). Quantification of the images showing (B) cell area, (C) actin fluorescent intensity, and (D) anisotropy of actin fibres. Quantification of the images to determine trend between primary cilium length and (E) cell area, (F) actin fluorescent intensity, and (G) anisotropy of actin fibres. (Student’s t-test, **p < 0.01, ****p < 0.0001. One-way ANOVA to determine trend between groups, #p < 0.05).

As cilia length has been linked with cell shape and actin contractility^53^, we next investigated whether the changes in primary cilia length may be associated with the recently identified estrogen withdrawal-induced changes in cell morphology^20^. Cells cultured under both estrogen and estrogen withdrawal conditions were analysed in terms of cell area and actin florescence intensity as indicators of intracellular tension/contractility. Cells cultured under estrogen withdrawal conditions were observed to be smaller and have a less developed actin cytoskeleton (Fig. 3A). Quantification of the images confirmed that estrogen withdrawal cells had a smaller cell area (328 ± 163 µm^2^), compared to estrogen cells (726 ± 497 µm^2^) (p < 0.0001, N = 3, n ≥ 92 cells per group) (Fig. 3B). Moreover, actin fluorescent intensity was significantly less intense in estrogen withdrawal cells (43,972 ± 35,246), compared to estrogen treated cells (31,492 ± 23,000) (p < 0.01) (Fig. 3C). However, no difference was seen in actin anisotropy between the estrogen and estrogen withdrawal cells (Fig. 3D). Next, we investigated whether the estrogen withdrawal-induced primary cilium elongation was associated with changes in cell area and actin cytoskeleton. Interestingly, a significant inverse relationship in cilium lengths could be seen with increasing cell area (p < 0.05, N = 6, n ≥ 5 cells per group) (Fig. 3E). An interesting inverse trend could also be seen between increasing primary cilium length and decreasing actin fluorescent intensity (p = 0.054, N = 6, n ≥ 11 cells per group). However, fibre anisotropy (p = 0.22, N = 6, n ≥ 25 cells per group) had no statistical association with primary cilium length (Fig. 3F,G).

**Figure 4:**
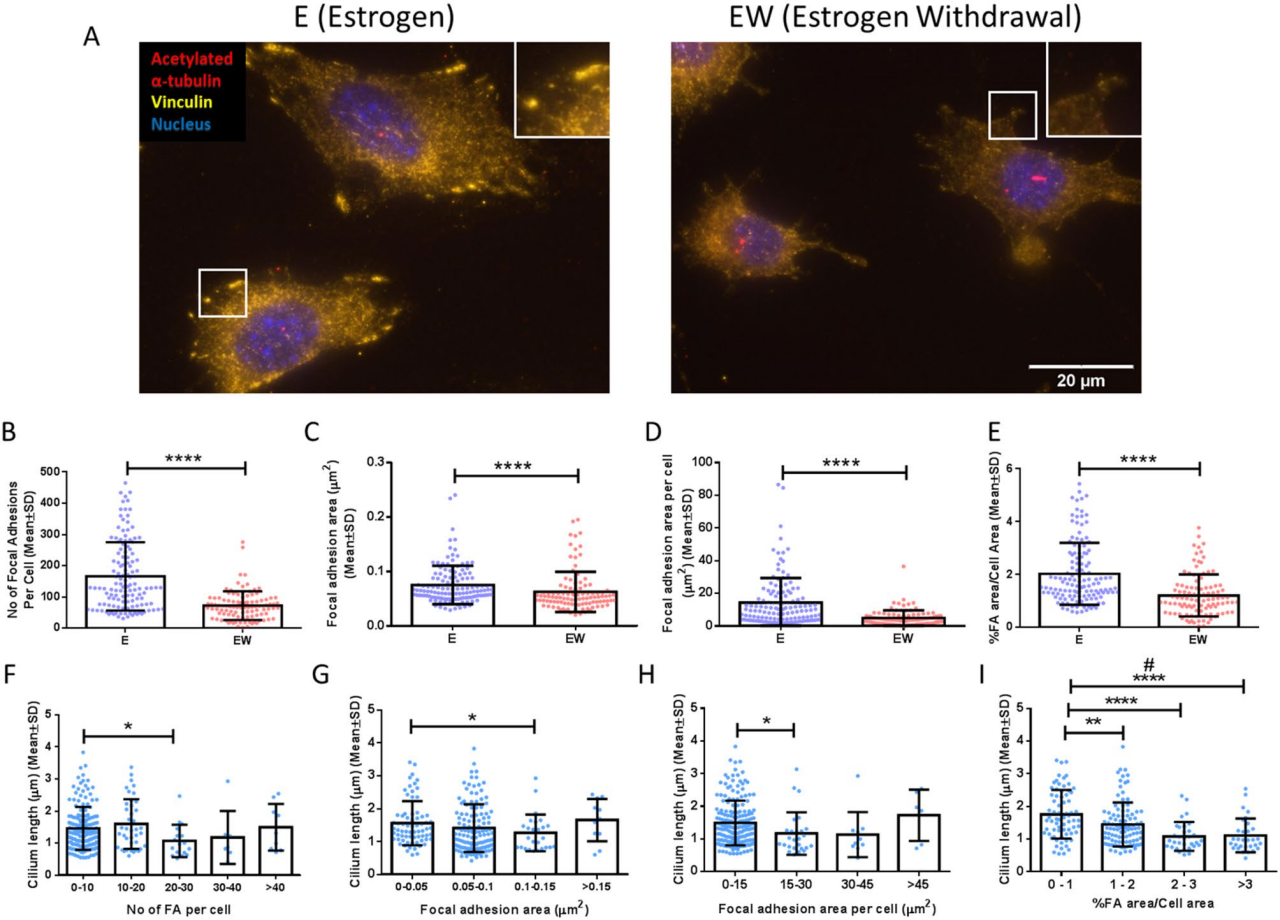
The effect of estrogen withdrawal on the role of the focal adhesion assembly in primary cilium dynamics. (A) Immunocytochemistry images showing acetylated α-tubulin, vinculin, and nuclei staining (N = 3, n ≥ 104 cells per group). Quantification of the images showing (B) number of focal adhesions per cell, (C) focal adhesion area, (D) focal adhesion area per cell, and (E) % focal adhesion area/cell area. Quantification of the images to determine trend between primary cilium length and (F) number of focal adhesion area per cell, (G) focal adhesion area, (H) focal adhesion area per cell, and (I) % focal adhesion area/cell area. (Student’s t-test, *p < 0.05, **p < 0.01, ****p < 0.0001. One-way ANOVA to determine trend between groups, #p < 0.05).

Following this, we next wished to investigate a potential association between estrogen withdrawal-induced changes in focal adhesion assembly seen previously with changes in ciliary structure^20^. Distinct vinculin-containing focal adhesion sites were clearly visible in the estrogen cells, but less so in the estrogen withdrawal cells (Fig. 4A). Quantification of the vinculin staining showed that estrogen withdrawal led to less focal adhesion sites (p < 0.0001), smaller focal adhesion sizes (p < 0.0001), and less focal adhesion area per cell (p < 0.0001) when compared to cells which were continually treated with estrogen (N = 3, n ≥ 104 cells per group) (Fig. 4B–D). Given the lower cell area seen in the estrogen withdrawal cells, the focal adhesion area per cell data was normalised to cell area to determine % focal adhesion area/cell area. This analysis further emphasised the reduction in the percentage focal adhesion area/cell area following estrogen withdrawal (p < 0.0001) (Fig. 4E) and demonstrated that the changes in focal adhesion assembly were not dependent on cell area. Next, we examined whether changes in primary cilium length were correlated with changes in focal adhesion assembly. A greater number of focal adhesions per cell (p < 0.05) (Fig. 4F), larger individual focal adhesions (p < 0.05) (Fig. 4G), and larger focal adhesion area per cell (p < 0.05) (Fig. 4H) were shown to lead to smaller cilium length. Interestingly, there was an inverse relationship between increasing % focal adhesion area/cell area and decreasing cilium length (p < 0.0001, N = 6, n ≥ 31 cells per group) (Fig. 4I).

Taken together, this data suggests that estrogen withdrawal disrupts focal adhesion assembly and intracellular actin contractility leading to an elongation of the primary cilium.

### Actin contractility inhibition and/or α_***v***_***β***_***3***_ antagonism mirror the effects of estrogen withdrawal on cell morphology and ciliary dynamics

To determine whether defined estrogen withdrawal induced effects on cell contractility and/or focal adhesion assembly directly affect ciliary dynamics, we next abrogated cell contractility via inhibition of the ROCK pathway (Y27632 treatment) or inhibited focal adhesion assembly via α_v_β_3_ antagonism in MLO-Y4 cells treated with estrogen. The α_v_β_3_ antagonist used here has been used previously on MLO-Y4 cells^20,50,51^. However, we found no record of the use of the actin contractility/ROCK inhibitor, Y27632, on MLO-Y4 cells. As such, a dosage study was performed with MLO-Y4 cells treated with 10 µM, 100 µM, and 1 mM Y27632 for 1 h. While stress fibres were still clearly visible in cells treated with 10 µM, they were no longer apparent in cells treated with 100 µM and 1 mM Y27632 (Supplementary Fig. 2A). Quantification of the images showed that 100 µM Y27632 treatment led to a smaller cell area (487 ± 265 µm^2^ vs 633 ± 406 µm^2^) (p < 0.01), less intense actin staining (18,274 ± 12,041 vs 42,180 ± 30,157) (p < 0.0001), and lower degree of actin fibre anisotropy (0.056 ± 0.039 vs 0.138 ± 0.073) (p < 0.0001), compared to control cells (N = 3, n ≥ 85 cells per group) (Supplementary Fig. 2B–D). 1 mM Y27632 treatment also led to a smaller cell area (p < 0.001), less intense actin staining (p < 0.0001), and lower degree of actin fibre anisotropy (p < 0.0001), compared to estrogen cells. Given the clear effect of 100 µM Y27632 treatment on the actin cytoskeleton, this dose was used going forward.

**Figure 5:**
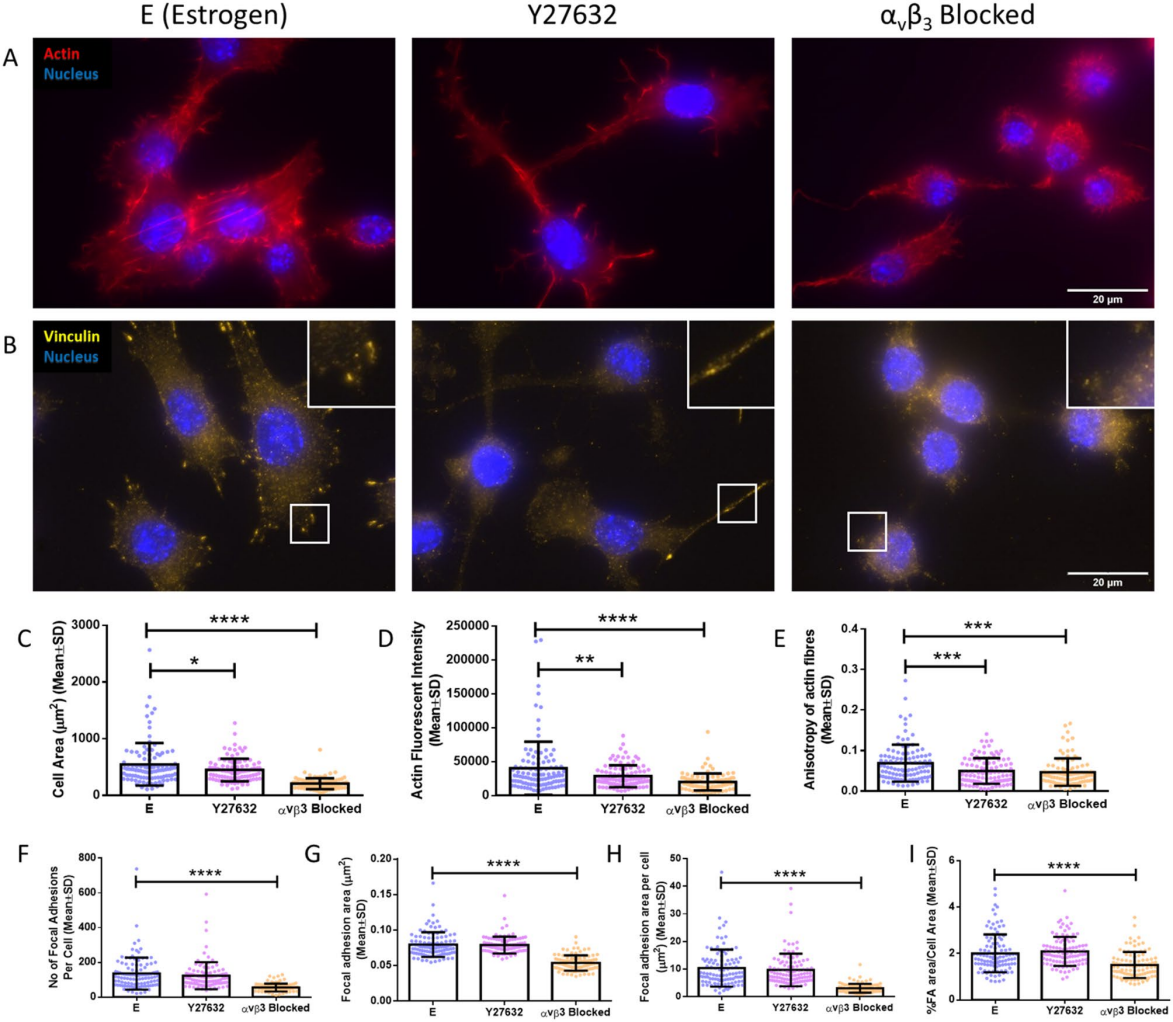
The effect of actin contractility inhibition (E + Y27632) and α_v_β_3_ antagonism (E + α_v_β_3_ antagonism) on the actin cytoskeleton and focal adhesion assembly. Immunocytochemistry images showing (A) actin and nuclei staining and (B) vinculin and nuclei staining (N = 3, n ≥ 95 cells per group). Quantification of the images showing (C) cell area, (D) actin fluorescent intensity, (E) anisotropy of the actin fibres, (F) number of focal adhesions per cell, (G) focal adhesion area, (H) focal adhesion area per cell, and (I) % focal adhesion area/cell area. (Student’s t-test, *p < 0.05, **p < 0.01, ***p < 0.001, ****p < 0.0001).

Inhibition of actin contractility following Y27632 treatment (ROCK inhibition) and α_v_β_3_ antagonism significantly altered cell morphology similar to that seen following estrogen withdrawal (Fig. 5A). Cells treated with Y27632 displayed long cell projections, as seen previously ^54^. Quantification of the images demonstrated that Y27632 treatment resulted in reduced cell area (449 ± 200 µm^2^ vs 547 ± 374 µm^2^) (p < 0.05), lower actin fluorescent intensity (28,575 ± 16,152 vs 40,162 ± 39,116) (p < 0.01), and lower degree of actin fibre anisotropy (0.049 ± 0.032 vs 0.069 ± 0.046) (p < 0.001), compared to estrogen control cells (N = 3, n ≥ 95 cells per group) (Fig. 5C–E), which is suggestive of a reduced cell contractility. Similarly, α_v_β_3_ antagonism led to a smaller cell area (206 ± 98 µm^2^ vs 547 ± 374 µm^2^) (p < 0.0001), lower actin fluorescent intensity (19,979 ± 12,421 vs 40,162 ± 39,116) (p < 0.0001), and lower degree of actin fibre anisotropy (0.047 ± 0.038 vs 0.069 ± 0.046) (p < 0.001), compared to estrogen controls.

As expected, distinct vinculin-containing focal adhesion sites were seen in estrogen control cells, which were absent following α_v_β_3_ antagonism (Fig. 5B). Interestingly, distinct focal adhesion sites were visible in Y27632 treated cells, indicating inhibition of actin contractility had little effect on focal adhesion assembly at the timepoints investigated in this study. Quantification of the images confirmed these observations with minimal effect seen following Y27632 treatment, while α_v_β_3_ antagonism resulted in reduced number of focal adhesion sites (55 ± 23 vs 135 ± 93) (p < 0.0001), smaller focal adhesion sites (0.053 ± 0.011 µm^2^ vs 0.079 ± 0.017 µm^2^) (p < 0.0001), less focal adhesion area per cell (3.01 ± 1.59 µm^2^ vs 10.35 ± 6.72 µm^2^) (p < 0.0001), and less % focal adhesion area/cell area (1.51 ± 0.56% vs 2.01 ± 0.82%), compared to control estrogen treated cells (N = 3, n ≥ 95 cells per group) (Fig. 5F–I).

**Figure 6:**
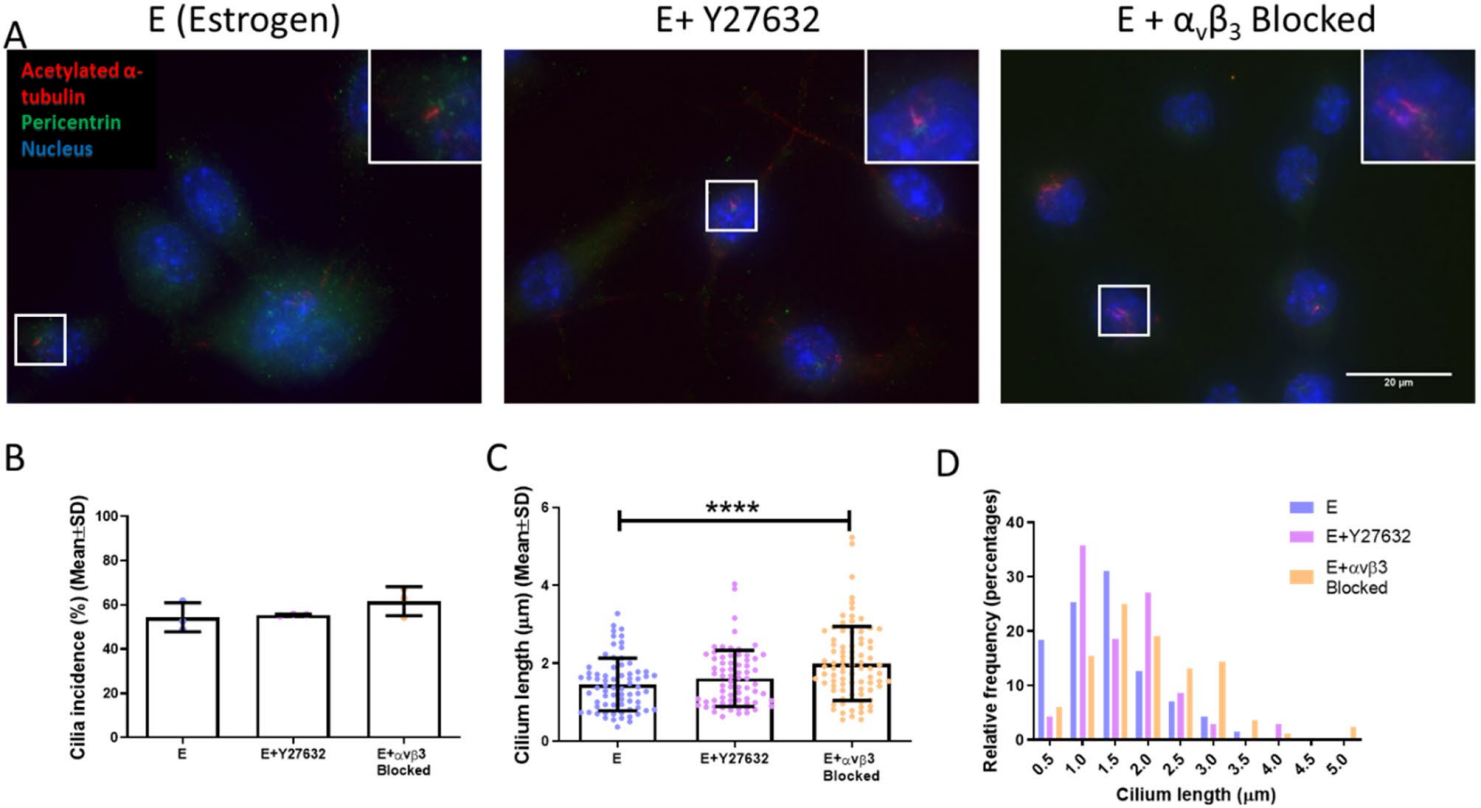
The effect of actin contractility inhibition (E + Y27632) and α_v_β_3_ antagonism (E + α_v_β_3_ antagonism) on MLO-Y4 primary cilium incidence and length. (A) Immunocytochemistry images showing acetylated α-tubulin, pericentrin, and nuclei staining (N = 3, n ≥ 70 cells per group). Quantification of the images showing (B) primary cilium incidence, (C) primary cilium length, and (D) distribution of primary cilia lengths. (Student’s t-test, ****p < 0.0001).

We next examined ciliary dynamics in conditions of reduced actin contractility and focal adhesion assembly. Abundant primary cilia staining was observed under all conditions, as seen by acetylated alpha-tubulin and pericentrin staining (Fig. 6A). Quantification of the images identified no statistical difference in primary cilia incidence between all conditions (Fig. 6B). However, a significant elongation of primary cilium length following α_v_β_3_ antagonism (1.996 ± 0.949 µm) was identified when compared to estrogen cells (1.456 ± 0.676 µm) (p < 0.0001, N = 3, n ≥ 70 cells per group) (Fig. 6C). A clear shift was displayed on a frequency histogram in the distribution of cilia lengths towards increased cilium length following α_v_β_3_ antagonism (Fig. 6D). Despite no statistical difference in primary cilium mean length following actin contractility inhibition (1.612 ± 0.720 µm) (Fig. 6C), the Y27632 treatment resulted in a shift to a bimodal distribution (Fig. 6D) of cilia length which would suggest that the Y27632 treatment affected a subpopulation of the cells analysed.

In summary, this data indicates that inhibited focal adhesion assembly and reduced actin contractility, as is seen following estrogen withdrawal, can lead to an elongation of the primary cilium.

### Actin contractility inhibition and/or α_***v***_***β***_***3***_ antagonism mirror the effects of estrogen withdrawal on cilia-associated and osteoclastogenic signalling

To further investigate whether changes in cilia-associated and osteoclastogenic signalling following estrogen withdrawal are associated with cell contractility and/or focal adhesion assembly, we next analysed Hh and *Rankl*/*Opg* gene expression following actin contractility inhibition and/or α_v_β_3_ antagonism.

**Figure 7:**
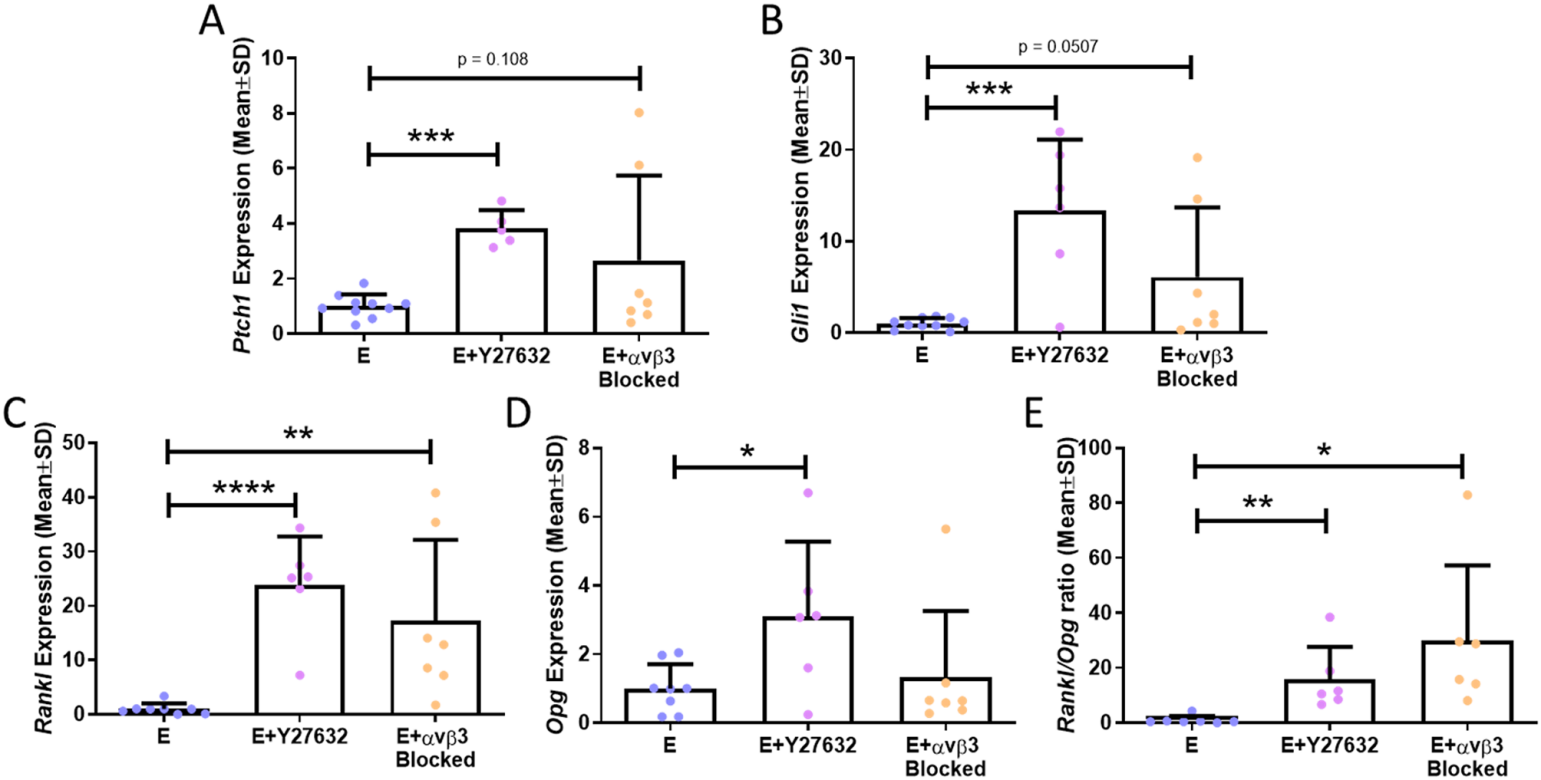
The effect of actin contractility inhibition (E + Y27632) and α_v_β_3_ antagonism (E + α_v_β_3_ antagonism) on Hh and osteoclastogenic signalling. qRT-PCR results of (A) *Ptch1* expression (N = 6–10), (B) *Gli1* expression (N = 6–10), (C) *Rankl* expression (N = 6–8), (D) *Opg* expression (N = 6–8), and (E) *Rankl*/*Opg* ratio (N = 6–7). (Student’s t-test, *p < 0.05, **p < 0.01, ***p < 0.001, ****p < 0.0001).

We first measured the Hh signalling markers, *Ptch1* and *Gli1* following Y27632 treatment and α_v_β_3_ antagonism and saw a significant 3.8-fold increase in *Ptch1* gene expression (p < 0.001) and 13-fold increase in *Gli1* gene expression (p < 0.001) following Y27632 treatment (N = 6–10). Similarly, α_v_β_3_ antagonism led to a threefold increase in *Ptch1* gene expression (p = 0.108) and sixfold increase in *Gli1* gene expression (p = 0.0507, N = 5–6) (Fig. 7A,B), both of which approach significance.

With regards to osteoclastogenic signalling, both Y27632 treatment (p < 0.001) and α_v_β_3_ antagonism (p < 0.01) resulted in increased *Rankl* expression (N = 5–6) (Fig. 7C). *Opg* expression was increased following Y27632 treatment (p < 0.05), but not following α_v_β_3_ antagonism (N = 6–8) (Fig. 7D). Taken as a ratio, there was an increase in the *Rankl*/*Opg* ratio following Y27632 treatment (p < 0.01) and α_v_β_3_ antagonism (p < 0.05) (N = 6–7) (Fig. 7E), indicating a shift towards osteoclastogenesis.

**Figure 8:**
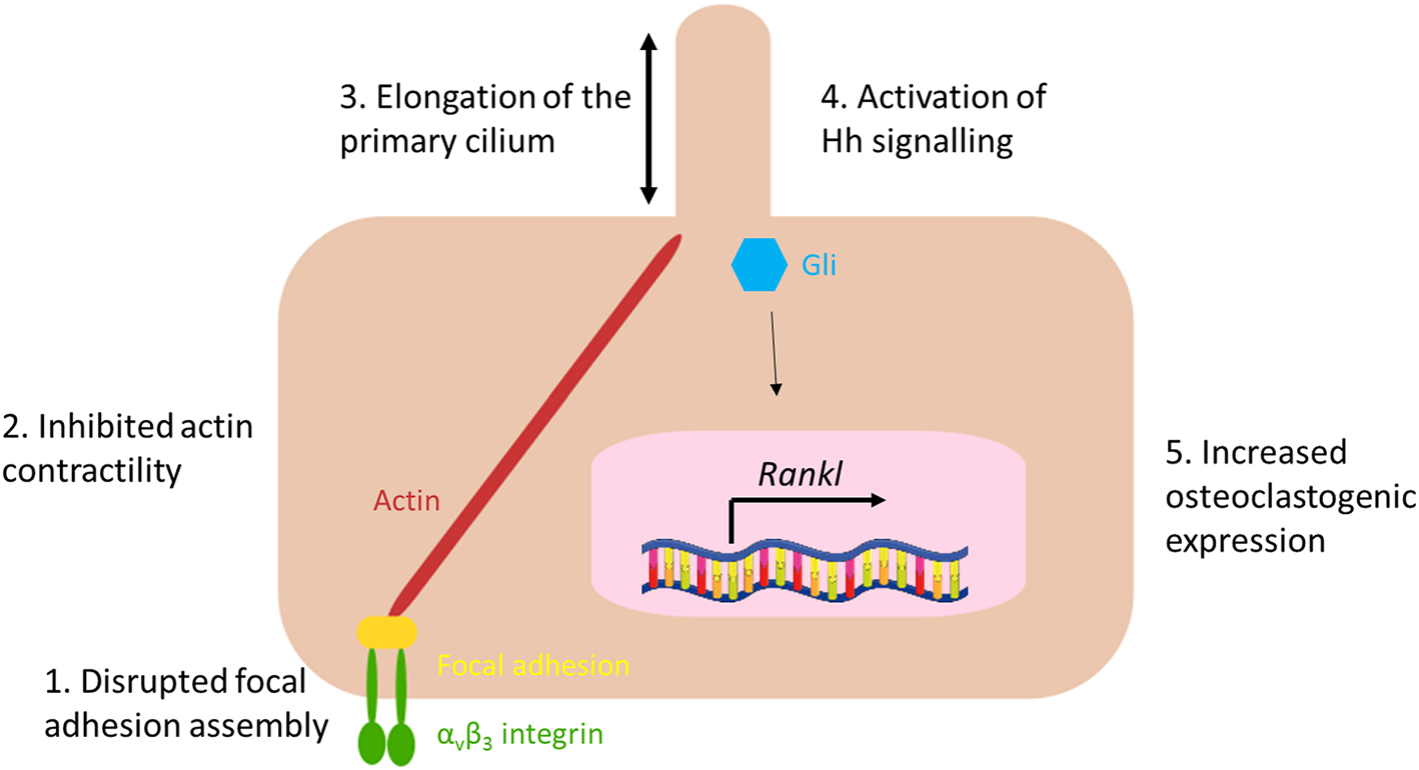
A schematic depicting the proposed effect of estrogen withdrawal, α_v_β_3_ antagonism, and actin contractility inhibition (Y27632) on the primary cilium and associated signalling. Following estrogen withdrawal, (1) focal adhesion assembly becomes disrupted and (2) actin contractility is inhibited, followed by (3) an elongation of the primary cilium, and resulting in (4) activation of Hedgehog signalling, and (5) an increased osteoclastogenic signalling as seen by increased *Rankl* gene expression. Focal adhesion assembly can also be directly disrupted using an α_v_β_3_ antagonist. Actin contractility can be directly inhibited using the ROCK inhibitor, Y27632. These direct interventions result in the same elongation of the cilium and resultant increases in Hh and osteoclastogenic signalling, as were found to occur under estrogen withdrawal.

In summary, this data indicates that estrogen withdrawal leads to inhibited focal adhesion assembly and reduced actin contractility, followed by an elongation of the primary cilium, which is associated with activation of Hedgehog signalling, and a shift towards osteoclastogenic paracrine signalling (Fig. 8).

## Discussion

Post-menopausal osteoporosis is a debilitating bone loss disease characterised by reduced levels of circulating estrogen. Here we demonstrated for the first time that estrogen withdrawal leads to an elongation of the primary cilium in osteocytes and increases in gene expression associated with Hh signalling, *Ptch1* and *Gli1*. The altered gene expression coincided with increases in *Rankl* expression seen previously^20^, which is consistent with previous studies linking the Hh and RANKL/OPG pathways^23^. We next investigated whether this alteration in cilia length and associated signalling was linked to alterations in FA assembly and actin contractility following estrogen withdrawal^20^. Significant trends were identified linking cilia elongation with reductions in cell area and % FA area/cell area, which indicates that cilia elongation may occur via a disruption of FA assembly and actin contractility following estrogen withdrawal. To further verify this, we inhibited FA assembly via α_v_β_3_ antagonism and demonstrated an elongated primary cilia and increased expression of Hh markers and *Rankl* expression. Moreover, we inhibited actin contractility via the ROCK inhibitor, Y27632, and found a shift towards an increased cilia length and significant increases in Hh markers and *Rankl* expression. On this basis we propose that the estrogen withdrawal conditions associated with post-menopausal osteoporosis lead to a disorganisation of α_v_β_3_ integrins and reduced actin contractility, which in turn can cause an elongation of the cilium. This alteration in ciliary dynamics may lead to an activation of the Hh pathway and osteoclastogenic paracrine signalling (Fig. 8).

It is interesting that we observe that estrogen withdrawal results in an elongation of the primary cilium and activation of Hh signalling. Primary cilium length has been shown to be altered in other pathological conditions, with cilium elongation arising in idiopathic scoliosis patients^63^, whereas shortened cilia have been identified to occur in alkaptonuria^41^ and Niemann-Pick C1 disease^64^. The primary cilium is involved in an entire class of pathological conditions known as ciliopathies. The ciliopathies mentioned above demonstrate altered primary cilia associated signalling, in particular Hh signalling, a key signalling pathway that occurs within the ciliary domain^39,65–67^. Primary cilium length has been shown to be associated with Hh signalling in chondrocytes^42^, whereby a reduced primary cilium length was associated with dysregulated Hh signalling^41,68^. A shorted primary cilium length has also been linked to dysregulated Hh signalling in fibroblasts and neurons^64,69^. In this respect, is interesting that our results demonstrate that estrogen withdrawal induces elongation of the primary cilium and results in an increase in the Hh markers, *Gli1* and *Ptch1*, highlighting a potential activation of Hh signalling via the cilium in the setting of post-menopausal osteoporosis. Hh signalling has been associated with expression of RANKL by osteoblasts^23^, and thus may also play an important role in the pro-osteoclastogenic phenotype seen in osteocytes following estrogen withdrawal. It must also be noted that PTH signalling may be an important factor in this increased osteoclastogenic phenotype, as when PTH1R was continuously activated in osteoblasts, RANKL expression was increased in mice with genetically activated Hh signalling^23^. However, in osteocytes where PTH1R was intermittently activated, antagonism of the Hh transcription factor, Gli1, led to increased *Rankl* expression^24^. Future studies involving the role of Hh signalling and activation of PTH1R in post-menopausal osteoporosis may offer novel therapeutic interventions.

Given the role of the actin cytoskeleton and focal adhesions in primary cilia dynamics, we next wished to determine whether the diminished focal adhesion assembly and disrupted actin cytoskeleton seen following estrogen withdrawal^20^ could be an effector of primary cilia elongation. We identified a significant trend of increasing cilium length with decreasing cell area, which is consistent with previous findings in retinal epithelial cells, where cells cultured on smaller micropatterns had an elongated primary cilium^53,69^. In this study, actin contractility was inhibited using the ROCK inhibitor, Y27632. Interestingly, one effect of Y27632 treatment was long cell projections, likely comprised of microtubule filaments ^54^. Moreover, similar to these studies^53,69^, here it is reported that disruption of actin contractility initiated a shift towards an increased cilia length, further highlighting the contribution of the actin cytoskeleton in ciliary dynamics. As actin contractility is generated via integrin-based attachments to the extracellular matrix, it was intriguing to identify an inverse relationship between % FA area/cell area and primary cilium length, whereby a smaller % FA area/cell area was associated with a larger cilium length. This relationship was further highlighted by the observation that osteocytes that underwent α_v_β_3_ antagonism had an elongated primary cilium in comparison to continuous estrogen treated cells. Taken together, this data contributes to a proposed mechanism whereby cilia elongation under post-menopausal conditions may occur indirectly via α_v_β_3_ integrin-containing focal adhesions and actin contractility. The exact mechanism of primary cilia elongation is complex, but the primary cilium has been shown to be regulated by and to regulate many cellular components, including the cytoskeleton and integrins. In particular, Pitaval et al. described a mechanism whereby spread cells with a highly contracted actin network, as is seen in our estrogen control groups^20^, orientate the nucleus-centrosome axis towards the ventral surface of the cell. In this position and in proximity with actin stress fibres, the centrosome’s ability to extend a cilium is inhibited, and this results in reduced ciliogenesis and cilia length as a result of contractility of the cell cortex^53^.

In the estrogen deficient environment of post-menopausal osteoporosis, the relative quantities of RANKL and OPG proteins are known to be altered^70,71^. We have previously reported that estrogen withdrawal leads to increases in *Rankl* gene expression, decreases in *Opg* gene expression, and an overall increase in the *Rankl*/*Opg* ratio in MLO-Y4 osteocytes^20^. We propose that estrogen withdrawal results in a disrupted focal adhesion assembly, an inhibited actin contractility, elongation of the primary cilium and increased Hedgehog signalling, which might play a role in the shift to pro-osteoclastogenic paracrine signalling, also reported here (Fig. 8).

A number of limitations must be considered. Firstly, our study involved immediate withdrawal of estrogen from a MLO-Y4 cell monolayer cultured after a period of estrogen accustomisation in cell culture media containing phenol red and FBS, which may contain basal estrogen (or derivates). However, the precise timeline of serum estradiol depletion in menopause is unknown. In humans, serum estradiol levels was shown to deplete over a 4 year period^72^, whereas in mice, serum estradiol levels were shown to be lower than normal controls 1 week after ovariectomy^73^. Further to this point, the levels of estrogen in the FBS were not measured and there may have been basal levels of serum-derived estrogen after withdrawal. However, all experiments were completed with the same batch of FBS to ensure a constant level of serum-derived estrogen was maintained for all groups and throughout the experiments. Secondly, the effect of the ROCK inhibitor, Y27632, was measured immediately following treatment. As such, sufficient time may not have been given to the cells to completely transduce the effect of inhibited actin contractility on primary cilium length. Moreover, while we report a relationship between cilia length, Hh signalling and osteoclastogenic signalling, further analysis is required to fully elucidate the underlying mechanisms by which these changes occur in osteocytes during estrogen deficiency. In particular, future studies should endeavour to understand the direct involvement of the cilium in estrogen deficiency related changes in paracrine signalling by osteocytes and whether this mechanism could be targeted therapeutically to treat post-menopausal osteoporosis. Lastly, this work was conducted using the MLO-Y4 cell line cultured in 2D. While this cell line is an established model that has been widely applied to study osteocyte biology, it may not capture the behaviour of primary osteocytes embedded in bone tissue in vivo. However, isolating primary osteocytes is a complex task and is associated with de-differentiation of the cells and a loss of the phenotypic behaviour of osteocytes in vivo, and so the use of a cell line was necessary. Future studies should explore this mechanism in environments that more closely recreate in vivo conditions, such as osteocyte-like cells cultured within 3D matrices and receiving physiologically relevant biophysical stimuli.

Our results show estrogen withdrawal conditions, associated with post-menopausal osteoporosis, result in an elongation of the primary cilium, increased expression of Hh markers, and increased expression of osteoclastogenic markers as seen in the *Rankl*/*Opg* ratio. Inhibition of actin contractility and α_v_β_3_ antagonism demonstrated similar results to that seen following estrogen withdrawal. Therefore, we propose that following estrogen withdrawal in osteocytes, α_v_β_3_ integrins become dysregulated and actin contractility is reduced leading to primary cilium elongation. This in turn, results in activation of the Hh pathway and increased osteoclastogenic paracrine signalling. This research further highlights the complex effect of estrogen withdrawal on osteocyte function and sheds new light on the possible mechanisms underpinning osteoclastogenic paracrine signalling.

## Supplementary Information

Supplementary Information.

## Data Availability

The datasets generated during and/or analysed during the current study are available from the corresponding author on reasonable request.
