# Supplementary material for: Estrogen withdrawal alters cytoskeletal and primary ciliary dynamics resulting in increased Hedgehog and osteoclastogenic paracrine signalling in osteocytes
Source: Sci Rep. 2021 Apr 29;11:9272. doi: 10.1038/s41598-021-88633-6 (PMC8085225; doi:10.1038/s41598-021-88633-6)
Supplement: Supplementary file 1 — Supplementary Information. [file 41598_2021_88633_MOESM1_ESM.docx]

**Estrogen withdrawal alters cytoskeletal and primary ciliary dynamics resulting in increased Hedgehog and osteoclastogenic paracrine signalling in osteocytes**

Ivor P. Geoghegan^1,2^, Laoise M. McNamara^1,2^, ^*^David A. Hoey^2,3,4,5^

^1^Mechanobiology and Medical Devices Research Group, Biomedical Engineering, College of Science and Engineering, National University of Ireland, Galway, Ireland

^2^Centre for Research in Medical Devices (CÚRAM), National University of Ireland, Galway, Ireland

^3^Trinity Centre for Biomedical Engineering, Trinity Biomedical Sciences Institute, Trinity College Dublin, Ireland

^4^Dept. of Mechanical, Manufacturing, & Biomedical Engineering, School of Engineering, Trinity College Dublin, Ireland

^5^Advanced Materials and Bioengineering Research Centre, Trinity College Dublin & RCSI, Dublin 2, Ireland

*Address for correspondence:

David A. Hoey,

Trinity Centre for Biomedical Engineering,

Trinity Biomedical Sciences Institute, Trinity College,

Dublin D02 R590, Ireland.

Email: dahoey@tcd.ie

**Supplementary Methods**

**Supplementary Table 1:** List of antibodies used

| **Antibody** | **Supplier** | **Reference** | **Dilution** |
| --- | --- | --- | --- |
| Acetylated α-tubulin | Abcam | ab26410 | 1:1500 |
| Pericentrin | Abcam | ab4448 | 1:1500 |
| Vinculin | ThermoFisher | 42H89L44 | 1:200 |
| Donkey anti-mouse Alexa Fluor 594 secondary antibody | Life tech | A21203 | 1:500 |
| Goat anti-rabbit Alexa Fluor 488 secondary antibody | Life tech | A11008 | 1:500 |

**Supplementary Table 2:** Primers used for qRT-PCR analysis

| **Gene** | **Sequence** | **Tm used (°C)** | **Primer Concentration** | **Amplicon size** |
| --- | --- | --- | --- | --- |
| *Ptch1* F | TGTGGCTGAGAGCGAAGTTT | 60 | 700 nM | 179 bp |
| *Ptch1* R | CACTCGTCCACCAACTTCCA |  |  |  |
| *Gli1* F | CAGCATGGGAACAGAAGGACT | 60 | 700 nM | 177 bp |
| *Gli1* R | GAGAGAGCCCGCTTCTTTGT |  |  |  |
| *Rankl* F | CCCATCGGGTTCCCATAAAG | 58 | 700 nM | 140 bp |
| *Rankl* R | AGCAAATGTTGGCGTACAGG |  |  |  |
| *Opg* F | GCCACGCAAAAGTGTGGAAT | 57.5 | 700 nM | 123 bp |
| *Opg* R | TTTGGTCCCAGGCAAACTGT |  |  |  |
| *Rpl13a* F | TACCAGAAAGTTTGCTTACCTGGG | 57.3 | 700 nM | 151 bp |
| *Rpl13a* R | TGCCTGTTTCCGTAACCTCAAG |  |  |  |

**Supplementary Results**


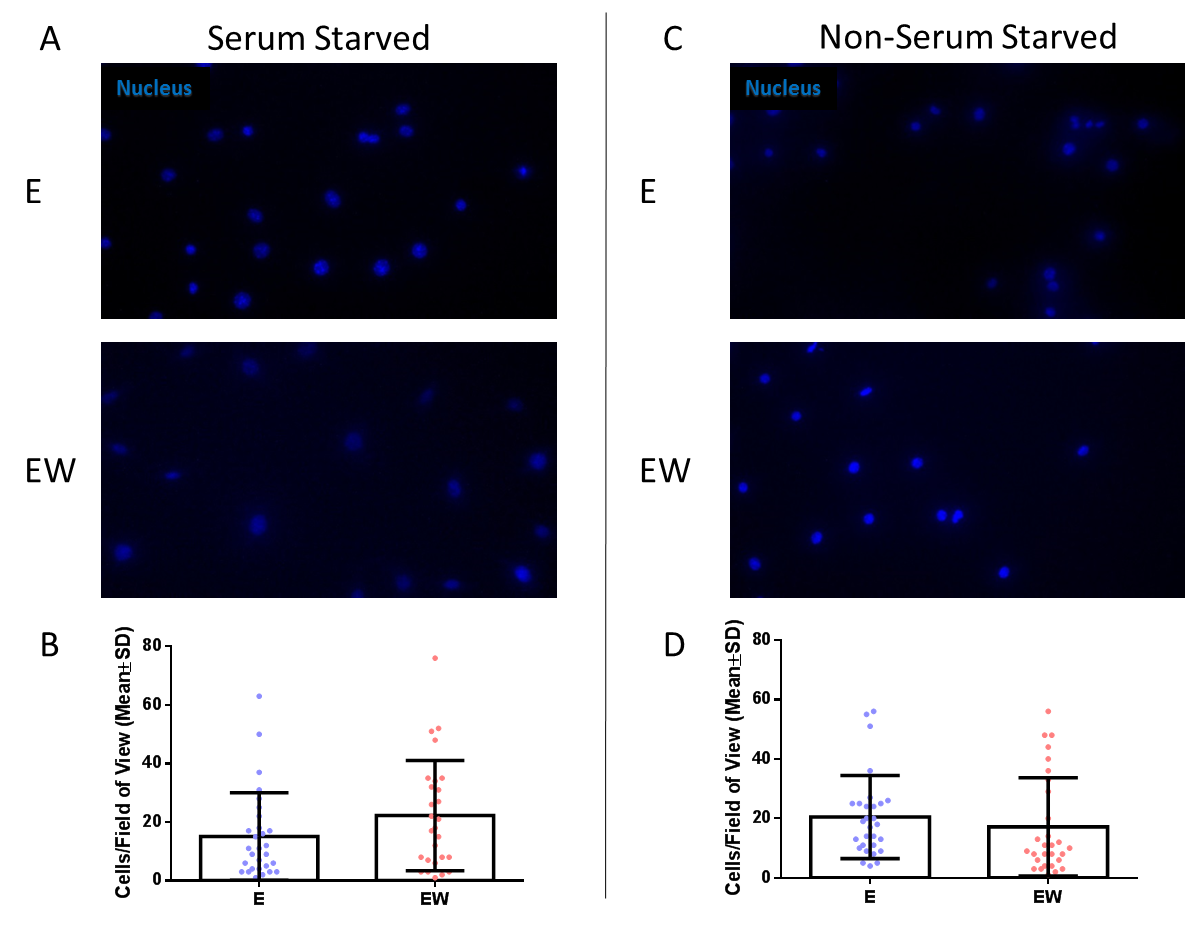


**Supplementary Figure 1:** The effect of estrogen withdrawal on MLO-Y4 cell number (N=3, n≥438 cells per group). (A) Immunofluorescent images showing nuclei staining for cell that underwent serum starvation for 48 h. (B) Quantification of the images showing the number of cells per field of view. (C) Immunofluorescent images showing nuclei staining for cells that did not undergo serum starvation. (D) Quantification of the images showing the number of cells per fields of view.


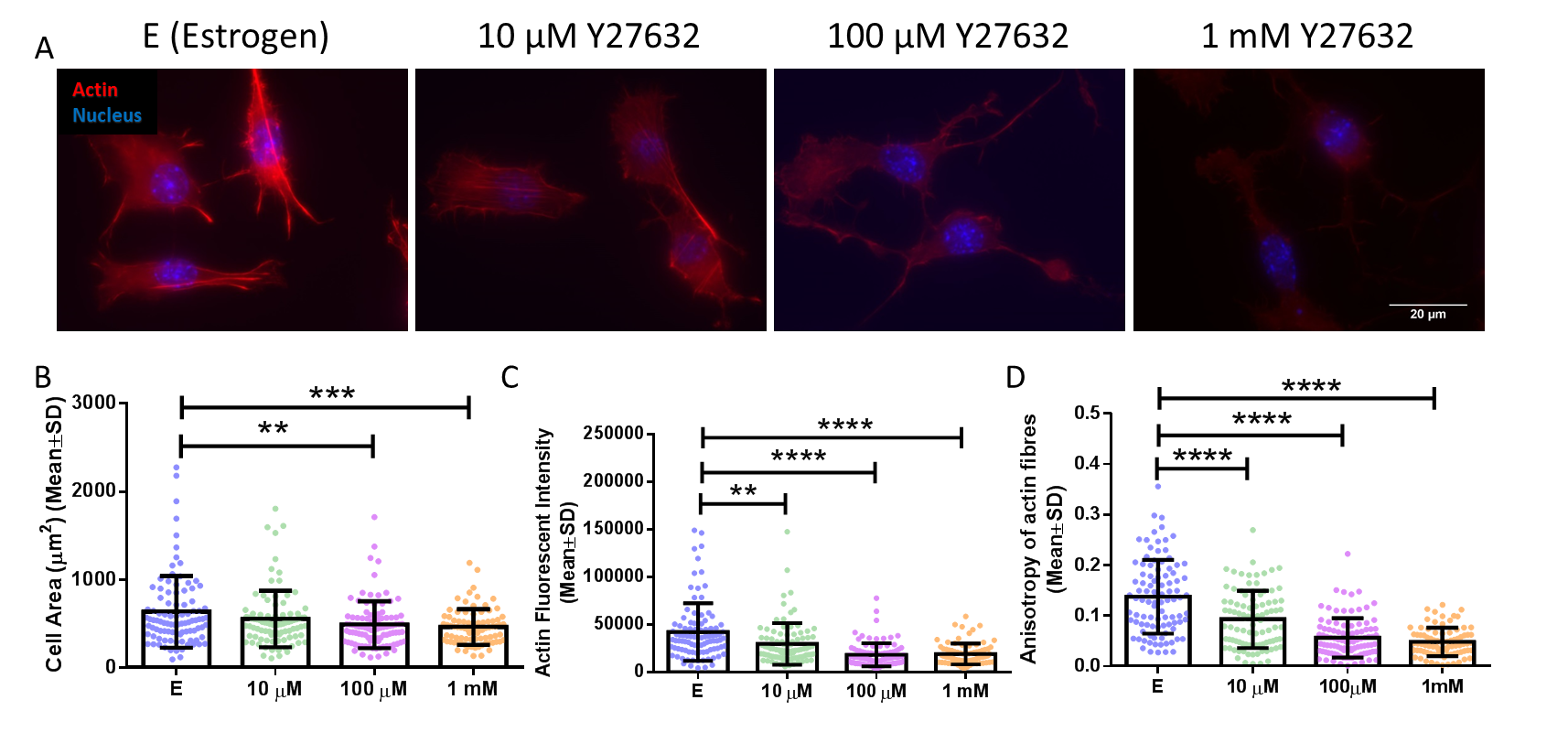


**Supplementary Figure 2:** The effect of different doses of the actin contractility inhibitor (Y27632) on MLO-Y4 cell size and actin cytoskeleton. (A) Immunofluorescent images showing actin and nuclei staining (N=3, n≥85 cells per group). Quantification of the images showing (B) cell area, (C) actin fluorescent intensity, and (D) anisotropy of the actin fibres. **p<0.01, ***p<0.001, ****p<0.0001)
